# Lysophosphatidic Acid Signaling in Cancer Cells: What Makes LPA So Special?

**DOI:** 10.3390/cells10082059

**Published:** 2021-08-11

**Authors:** Pravita Balijepalli, Ciera C. Sitton, Kathryn E. Meier

**Affiliations:** Department of Pharmaceutical Sciences, College of Pharmacy and Pharmaceutical Sciences, Washington State University, Spokane, WA 98686, USA; pravita.balijepalli@wsu.edu (P.B.); ciera.sitton@wsu.edu (C.C.S.)

**Keywords:** lysophosphatidic acid, G protein-coupled receptors, cancers

## Abstract

Lysophosphatidic acid (LPA) refers to a family of simple phospholipids that act as ligands for G protein-coupled receptors. While LPA exerts effects throughout the body in normal physiological circumstances, its pathological role in cancer is of great interest from a therapeutic viewpoint. The numerous LPA receptors (LPARs) are coupled to a variety of G proteins, and more than one LPAR is typically expressed on any given cell. While the individual receptors signal through conventional GPCR pathways, LPA is particularly efficacious in stimulating cancer cell proliferation and migration. This review addresses the mechanistic aspects underlying these pro-tumorigenic effects. We provide examples of LPA signaling responses in various types of cancers, with an emphasis on those where roles have been identified for specific LPARs. While providing an overview of LPAR signaling, these examples also reveal gaps in our knowledge regarding the mechanisms of LPA action at the receptor level. The current understanding of the LPAR structure and the roles of LPAR interactions with other receptors are discussed. Overall, LPARs provide insight into the potential molecular mechanisms that underlie the ability of individual GPCRs (or combinations of GPCRs) to elicit a unique spectrum of responses from their agonist ligands. Further knowledge of these mechanisms will inform drug discovery, since GPCRs are promising therapeutic targets for cancer.

## 1. Introduction

Lysophosphatidic acid (LPA) refers to small phospholipids with a single fatty acid substituent with an acyl or alkyl linkage to the glycerol backbone. An abundant species in mammals, usually utilized experimentally in cell culture, is 18:1 (oleoyl)-LPA. LPA stimulates the proliferation of a wide variety of cell types and is one of the significant growth factors present in the sera used for cell culture. It is therefore not surprising that the role of LPA in cancer has been closely studied and that LPA antagonists have been developed and tested as therapeutic agents for cancer. The application of LPA antagonists in cancer treatment is reviewed in another article of this Special Issue [1].

LPA exerts most of its effects by acting as an agonist of G protein-coupled receptors (GPCRs), termed LPA receptors (LPARs), which were first described as binding sites in 1994 [2], and cloned in 1996 [3]. LPARs are among the many GPCRs being studied as potential targets for therapies for a myriad of human disorders, including cancer [4,5]. Currently, the LPAR family includes six members, designated LPAR1–LPAR6 [6,7,8,9,10,11,12,13,14,15]. The LPAR nomenclature (LPAR 1–6) was established by the HUGO Gene Nomenclature Committee (https://www.genenames.org/data/genegroup/#!/group/205; accessed 10 August 2021). GPR87/95, another GPCR, is a putative LPAR [16]. In addition, several non-GPCR proteins have been reported to serve as receptors for LPA. PPARγ, a member of the PPAR family of nuclear receptors, can mediate responses to LPA [17,18]. Receptor for advanced glycation end products (RAGE) can also mediate LPA actions, including proliferation in lung and breast cancer cells [19]. The characteristics of the various LPARs, and the roles that they play in physiological and pathophysiological processes, have been reviewed in detail [20]. LPA production, LPA degradation, and/or LPA receptor expression can be altered in cancer cells, thereby helping to support tumor growth [20,21,22,23,24,25]. This review will focus on LPAR signaling, and not on LPA generation. While increases in receptor expression are often advantageous to the growth and survival of cancer cells, it should be noted that LPA receptors (LPARs) are ubiquitously expressed in both normal and transformed cells and are thus available for therapeutic targeting in many different situations. The expression levels of individual receptors do, however, vary between different types of cells and tissues. This provides an opportunity to differentially target LPARs in cancer cells by receptor-selective antagonists. The widespread expression of LPARs means that increased LPA production, or decreased LPA degradation, can result in effects on many different organs and physiological processes [18,26].

LPA is a growth factor that also stimulates cell migration and can therefore contribute to both cancer progression and metastasis [27,28]. LPARs are coupled to the heterotrimeric G proteins G_s_, G_i/o_, G_q/11_, and G_12/13_, depending on the receptor. LPARs also alter cellular functions via the small GTPases Ras, Rac, and Rho to produce diversified effects. LPA is particularly efficacious at activating Rho [29]. LPARs as potential therapeutic targets and the LPA “axis” in cancer have been reviewed on multiple occasions over the years [1,15,18,20,30,31,32], including in the current Special Issue [33].

To date, the focus on the mechanisms by which LPA stimulates the proliferation of cancer cells has, understandably, been on G proteins. Each LPAR activates its own portfolio of signaling effectors, but in general, LPARs facilitate cancer cell proliferation and survival via G_q_, G_i_, and/or G_12/13_. LPARs stimulate cell migration and invasion mainly via G_i_, and/or G_12/13_. In this respect, LPARs are no different from hundreds of other GPCRs. However, the prominent effects of LPA on both cell proliferation and migration raise the question: What makes LPA so special? The answer to this question is still evolving. To answer it fully, we must consider emerging concepts of how GPCRs transmit their signals. The discussion will shed light on the need for a more holistic approach to the evaluation of the roles of LPARs and other GPCRs in cancer.

## 2. Highlighted Aspects of LPA Signaling

Two relatively understudied aspects of LPAR-mediated signaling have the potential to provide more nuance to the mechanisms by which LPA stimulates cancer cell growth.

First, LPARs were key to the initial discovery that GPCRs can induce transactivation of the epidermal growth factor receptor (EGFR) [34]. This study, carried out in kidney and bladder cancer cell lines, established the paradigm in which LPA (and other GPCR agonists) can induce the release of EGFR ligands that activate EGFR in an autocrine manner. A subsequent report confirmed that LPA can induce the release of multiple EGFR ligands in non-cancerous epithelial cells, but also showed that, in the same cells, the ERK pathway could be activated by different growth factor receptors in an EGFR-dependent manner through multiple mechanisms [35]. These studies show that enhancement of EGFR activity and activation of mitogenesis do not occur through only a single pathway. The extent to which EGFR transactivation is responsible for LPA-induced mitogenic responses in various cell types remains to be determined. Examples will be addressed in subsequent sections of this review.

**Figure 1:**
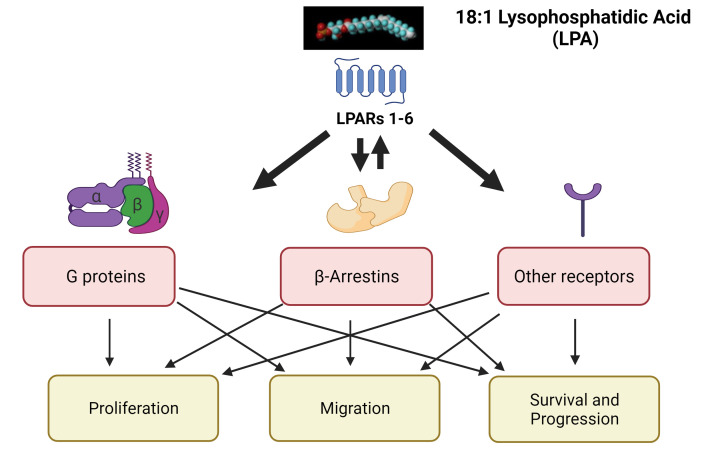
Pathways for LPA-mediated signal transduction in cancer cells. Although 18:1-LPA is depicted here, it is not the only species of LPA with biological activity. LPA can activate any of the six confirmed LPA receptors (LPARs) in the plasma membrane. These receptors interact with G proteins, β-arrestins, and/or other membrane receptors to transduce signals that enhance cell proliferation, migration, and survival. β-Arrestins also play a role in desensitizing receptor activity via GPCR internalization. Partnering receptors can either enhance LPA production and LPAR activity (e.g., EGFR, CD97) or inhibit LPAR activity (e.g., FFAR4). This figure was made using www.biorender.com; accessed 4 August 2021.

Second, most studies examining the mechanism of action of LPA in cancer cells have involved the study of downstream signaling pathways, rather than events occurring at the receptor level. However, agonist-bound LPA receptors are phosphorylated by G protein-coupled receptor kinases (GRKs) and can then recruit β-arrestins, scaffolding proteins that are now known to function in various capacities from receptor internalization to signaling [36,37]. The contributions of β-arrestins to the responses initiated by individual LPARs have received relatively little attention. Protein–protein interactions between LPARs and other signaling partners (e.g., EGFR) are also underexplored. These topics will be considered below. Figure 1 depicts the types of signaling pathways, downstream of LPAR activation in cancer cells, to be addressed in this review. 

## 3. Scope

In this review, we consider the signaling pathways and molecular mechanisms involved in the responses to LPA in cancer cells, with the goal of developing future therapeutics. Background references will be provided for perspective, but the focus will be on recently published results. Only GPCR-mediated actions will be considered herein. The focus of this review is on mechanisms of LPAR signaling, and not on autocrine/paracrine mechanisms of LPA generation. The role of autotaxin in generating extracellular LPA has been abundantly reviewed and will not be revisited here [21,23,38,39]. Similarly, LPA degradation has been covered in general reviews concerning LPA action and will not be covered here. Geraldo and colleagues [25] published a recent review that addresses both LPA production and degradation.

The initial sections of this review are organized with respect to different types of cancer. The analysis focuses on recent publications that specifically concern signaling mechanisms, since many excellent reviews have been published previously concerning the generation and cellular activities of LPA. The concluding sections will discuss emerging molecular aspects of LPA signaling, along with the remaining knowledge gaps.

## 4. LPA Signaling in Specific Cancer Types

### 4.1. Prostate Cancer

Since LPA signaling has been studied extensively in prostate cancer cells [40], prostate cancer provides many examples of molecular mechanisms of LPA action. An early study from our group demonstrated that LPA stimulates the proliferation of human prostate cancer cell lines [41]. LPAR1, LPAR2, and LPAR3 are expressed at varying levels in three commonly studied human prostate cancer lines, PC-3, DU145, and LNCaP [42,43,44].

Early on, LPAR1 was identified as the receptor mediating many of the stimulatory effects of LPA on the proliferation and migration of prostate cancer cells [45]. This conclusion was reached through studies of LPAR expression and response [46], use of pharmacological LPAR antagonists [31,47,48], and LPAR1 knockdown [44,48,49]. LPAR1 and LPAR3 have been implicated in the induction of reactive oxygen species (ROS) via protein kinase C in PC-3 cells [50]. In androgen-dependent LNCaP cells, implantation of LPAR1 receptor increases LPA responsiveness of the cells and promotes nuclear localization of the androgen receptor [45].

Interestingly, one report suggested that LPAR1 expression in androgen receptor-expressing prostate cancer cells is regulated in an androgen-dependent manner [51]. In comparison with LPARs 1–3, little is known about LPAR4 and LPAR5 as they are expressed at low levels in prostate cancer cells [52].

Genc and co-workers [53] observed that LPA suppresses autophagy in PC-3, DU145, and LNCaP cells that have been serum-starved. They also reported that LPA activates both the ERK and mTOR pathways in serum-free and serum-containing media; activities of both kinases were required for the suppression of autophagy. These results are particularly notable because serum starvation is commonly used to deplete cells of LPA (a component of serum) prior to experiments examining LPA effects. 

The cytoskeletal effects of LPA are prominent in cancer cells. LPAR1 activation was reported to stabilize acinar morphology and regulate cytoskeletal organization in prostate cancer cells [54]. Transcriptome analyses revealed that lysine-specific demethylase 1 (LSD1) promotes LPAR6-dependent migration in androgen-independent prostate cancer cells [55]. The effects of LPA on actin-related proteins such as vasodilator-stimulated phosphoprotein (VASP) were also observed to play a role in cell motility and metastases in prostate cancer cells by stimulating the formation of lamellipodia [56].

Further downstream, the effects of LPA on gene transcription propagate signals in additional directions. Lin and colleagues [57] reported that LPA induces the transcription of mRNA encoding VEGF-C, which contributes to lymph angiogenesis in prostate cancer cells. High glucose levels have been observed to increase VEGF-C transcription via an LPAR1/LPAR3-Akt-Ros-LEDGF signaling axis [58]. Shin and colleagues [48] implicated the induction of Krupper-like factor 5 in LPA-induced proliferation and migration in PC3 cells. LPA induces the production of CCN1, a matricellular protein involved in adhesion signaling [59].

Invasive tumor cells produce stromal growth factors, including cytokines and lipids, that support growth and invasion. LPA activates Akt/Rho in PC-3 cells, thereby resulting in the formation of invadopodia that aid metastasis [60]. Bone metastasis is a common occurrence in advanced prostate cancer; hence crosstalk between cancer cells and bone microenvironment is a critical aspect. LPA can stimulate osteoclasts indirectly by changing local interleukin levels and by directly stimulating osteoclast differentiation [61]. In this study, using mouse calvarium xenografts, LPA (injected intraperitoneally) stimulated PC-3 cells to release osteoclastogenic cytokines (including IL-6), enhancing bone destruction.

Emerging studies also reveal β-arrestin as an important signaling molecule in prostate cancer. Knockdown of arrestin-3 (β-arrestin-2) blocks LPA-induced proliferation in PC-3 and DU145 cells [49]. One of the best characterized pathways for arrestin-mediated proliferation is a complex functional interaction between β-arrestin and c-Src, which can mediate crosstalk with EGFR [62,63]. 

While there are few studies of protein–protein interactions involving LPARs, one published example concerns prostate cancer cells [64]. In this report, the authors showed that LPAR1 can heterodimerize with CD97, a GPCR involved in adhesion. CD97 signals via G_12/13_ to activate Rho. The authors implicated heterodimerization in the progression of prostate cancer to bone metastasis.

### 4.2. Breast Cancer

Multiple reviews have addressed the role of LPA in breast cancer [65,66,67]. It has been reported that breast cancer cells express elevated levels of LPAR1, LPAR2, and LPAR3 and that these LPA receptors are involved in stimulating tumorigenic and metastatic cascades via Wnt, MAPK, and PI3K pathways. Interestingly, adipose stroma has been reported to exhibit a unique expression profile for LPARs and autotaxin in breast cancers [68].

Regarding specific receptors, overexpression of LPAR1 and LPAR2 has been observed across breast cancers, as reported by several research groups [69,70,71,72]. A particularly important study by Liu and colleagues [38] showed that overexpression of LPAR1, LPAR2, or LPAR3 in transgenic mice could induce mammary tumors. Although there were differences in the mammary lesions observed with the different receptors, this study suggests that LPARs 1–3 have overlapping functions.

Similar to the situation in prostate cancer cells, LPA enhances migration in breast cancer cells [73,74]. In MCF10CA1a cells, LPA increases cell contractility via an LPAR1/Rho/ROCK pathway and promotes cell motility via G_i/o-_ and G_q/11-_mediated signaling [75].

In an opposing example, Tao and co-workers [76] explored the role of LPAR6 in breast cancer. Their results suggested that LPAR6 may be a tumor suppressor. The expression of LPAR6 was suppressed in breast cancer tissue, and low expression was correlated with poor prognosis. Knockdown of LPAR6 enhanced the proliferation and migration of the ZR-75-1 cell line. Computational analysis of gene expression data identified potential downstream targets of LPAR6. 

One group investigated the ability of breast cancer cells to secrete cytokines that enhance osteoclast genesis [77]. The investigators focused on the production of IL-8 and IL-11 in response to LPA. Signaling proteins implicated in IL-8 secretion were ROCK, PKCμ, PI3K, and NFκB. The involvement of LPAR1 and LPAR2 was inferred but not tested. On a more translational note, a pharmacological agent that is a pan-LPAR antagonist and autotaxin inhibitor was shown to inhibit breast cancer cell migration [78]. In the same study, this compound suppressed MDA-MB-231 tumor growth in a xenograft model in mice. Another group reported that inhibition of autotaxin in a syngeneic orthotopic mice model of breast cancer reduced LPA-induced lung metastasis [79].

### 4.3. Ovarian Cancer

LPA, its receptors, and its role as a biomarker have been extensively studied in ovarian cancer and have been reviewed [80,81,82]. The role of LPA in ovarian cancer was first reported in 1995 [83]. In early work, Goetzl and co-workers [84] investigated the expression and function of Edg family receptors (including those activated by sphingosine 1-phosphate) in primary ovarian cultures and ovarian cancer cell lines, with a focus on LPAR2, which was upregulated in cancer cells. Primary ascites-derived ovarian tumor cells predominantly express LPARs 1–3 [85]. Expression of LPAR2 and LPAR3 has been correlated with increased cell aggressiveness in ovarian cancer [86]. One group studied LPAR expression and LPA-induced migration in 13 ovarian cell lines [87]. They concluded that LPAR1 expression is higher in more metastatic cell lines, that only the silencing of LPAR1 reduces LPA-induced invasion, and that LPAR1 is more highly expressed in more advanced stages of human cancer. By contrast, others showed that LPAR2 knockdown reduced p-ERK levels in ovarian cancer cell lines. On a similar note, knockout of LPAR2 resulted in reducing LPA-induced migration in SKOV3 cells [81,88]. LPAR1 and LPAR2 have been reported to show redundant signaling since double knockdown of both the receptors resulted in substantial lethality in the cells [89]. 

LPA can mediate increases in tumor-associated macrophages through the AKT/mTOR pathway, which in turn leads to tumor promotion [90]. Inhibition of LPA-induced survival and proliferation in SKOV3 cells with VPC32183 also enhanced the response to chemotherapeutic drugs such as Taxol and increased cell death [91].

Park and colleagues [92] reported that LPAR1 and LPAR2 induce the migration of OVCAR-3 cells via G_12/13_/RhoA, in a pathway requiring the phosphorylation of ezrin/radixin/moesin proteins. LPAR3/G_13_ signaling is also implicated in YAP-mediated long-term migration of human ovarian cancer cells [93].

SIRT1, a protein that acts as an oncogene in many cancers, regulates epithelial-to-mesenchymal transition (EMT) in ovarian cancer cells [94]. LPA was found to downregulate SIRT1, causing HIF1α to remain in its acetylated functional form that triggers invasiveness [95]. LPAR3 was implicated in this response. LPA induces cyclooxygenase (COX-2) via LPAR3, leading to greater production of prostaglandins [96]. 

LPA can stimulate the proliferation, migration, and invasion of CAOV3 cells and upregulate the expression of the cytokine CXCL12 [97]. These findings suggest that CAOV3 cells can secrete CXCL12 in an autocrine manner. CXCL12 can regulate directional migration, invasion, and angiogenesis of tumor cells and is found at high levels in the ascites fluid of ovarian cancer patients. However, another group found that LPA stimulated proliferation but not CXCL12 production in CAOV3 cells [98]. Other groups have reported that LPA induces IL-8, an angiogenic factor that contributes to ovarian cancer metastasis in ovarian cancer cell lines [99].

Elevated levels of LPA have been linked to the metastasis and dissemination of tumor cells from multicellular aggregates. LPA plays a role in promoting the differential implantation of mesenchymal-type metastasis cell clusters in vivo [100]. When OvCa429, DOV13, and SKOV3ip cells were incubated in vitro with LPA, aggregation was reduced in all three cell lines. Inhibition of calcium mobilization, by linoleic acid, significantly reduced LPA-induced formation of focal adhesions in SKOV3 cells [101].

Oyesanya and colleagues [102] investigated the role of EGFR transactivation in LPA-induced activation of transcription factors. Specifically, they examined which G protein responses required EGFR. They concluded that Gi-dependent responses to LPA require EGFR transactivation, but that G_q- _or G_12/13-_dependent responses (e.g., NF-kB activation) do not. Another group provided evidence that LPA induces EGFR transactivation in ovarian cancer cells downstream of both G_i_ and G_13_.

Interesting work has addressed the role of LPA in ovarian cancer stem cells. In these cells, LPA upregulates the zinc transporter Zip4 through PPARγ [103].

The diagnostic and therapeutic potential of LPA has been well studied in the context of ovarian cancer, although this is not the focus of the current review. For example, one case study reported high levels of LPA as a prognostic biomarker in ovarian cancer patients [104]

### 4.4. Melanoma

Several groups have reported data concerning LPARs and their roles in melanoma. B16F10 cells predominantly express LPAR2, LPAR5, and LPAR6 [105]. The chemorepellent effect of LPA on B16 melanoma cells was investigated, along with its pro-invasive role [106]. LPAR5 was determined to mediate this response, via cAMP generation, based on knockdown experiments. Another group reported a role for LPAR5 in chemoresistance in A375 cells, where chemotherapy agents induced the expression of LPAR5 [107]. These investigators later observed that LPAR2 activation enhances chemoresistance in the same cell line [108]. 

In experiments utilizing the WM239A melanoma cell line, Susanto and colleagues [109] described an interesting scenario in which the cells self-generate chemotactic LPA gradients via breakdown of local extracellular LPA by the lipid phosphatase LPP3. LPAR1 is the receptor that appears to be involved in the chemotactic response, according to previous work by the same group [110].

### 4.5. Endometrial Cancer

The role of LPAR2 in the invasion of endometrial cancer cells has been investigated [111]. Using LPAR2 knockdown, the investigators demonstrated a specific role for this receptor in the invasion, but not adhesion, of Hec-1A cells. Although LPA activates MMP-7, this protease does not appear to be responsible for LPA-induced cell invasion [112]. A subsequent study compared the mRNA expression levels of LPAR1, LPAR2, and LPAR3 in human endometrial cancer samples to those in adjacent non-cancerous tissue [113]. The results showed that expression of all three receptors was increased in cancerous tissue. LPAR2 was the predominant receptor in Hec-1A cells, consistent with the work discussed above. The investigators examined LPA-induced proliferation in Ishikawa endometrial carcinoma cells and identified a role for the ERK MAPK pathway in the response.

### 4.6. Pancreatic Cancer

LPA is generally mitogenic in pancreatic cancer, and LPAR expression levels are increased in this disease. The critical roles of LPARs in pancreatic cancer have been reviewed [114]. LPA signaling has been studied in the PANC-1 human pancreatic cell line [115]. The investigative team generated cisplatin-resistant PANC-1 cells; these were more motile and invasive and expressed higher mRNA levels of LPAR1 and LPAR3. Knockdown of LPAR1/LPAR3 reduced the motility and invasion of the cisplatin-resistant cells. LPA stimulates a transient mitogenic response in MDA Panc-8 cells via Gα13 [116].

In PANC-2 cells, which express LPARs 1–3, LPA activates FAK and paxillin [117]. These responses were linked to LPA-induced cell motility. Stähle and co-workers [118] found that LPA was not mitogenic for PANC-1 and BxPC-3 cells, but stimulated migration via a G_i/o_/ERK-dependent mechanism. Based on the results discussed above, there is some inconsistency regarding the ability of LPA to induce proliferation of pancreatic cancer cells, although enhancement of motility is a prominent finding.

LPA robustly stimulates RhoA activation, which is followed by the induction of myosin II-dependent contractility [119]. An essential part of actin remodeling is the nucleation of the filaments. These filaments branch with the help of the Arp2/3 complex, mediated by nucleation promoting factors (NPFs) of which N-WASP is a critical member. Juin and colleagues [120] have established that N-WASP contributes to chemotaxis toward LPA. Interesting perspectives have been provided about the functionality of N-WASP and the LPA axis in pancreatic cancer [121].

### 4.7. Hepatocellular Carcinoma

The LPA axis is particularly important in hepatocellular carcinoma (HCC) [122,123]. Several LPARs have been implicated in HCC. LPAR1 mediates LPA-induced migration and MMP-9 expression in HCC cell lines [124]. Higher mRNA levels of LPAR1, LPAR3, and LPAR6 in HCC tissues were correlated with worse prognosis in resected human livers [122]. There is a correlation between higher mRNA levels of LPAR2 and more poorly differentiated HCC tumors [125]. In addition to LPAR2, elevated levels of LPAR6 are associated with liver malignancy [126]. Overexpression of LPAR6 is associated with severe clinical outcomes in patients with HCC [127].

LPA enhances the trans-differentiation of peritumoral tissue fibroblasts [128]. The LPA antagonists 4-methylene-2-octyl-5-oxotetra-hydrofuran-3-carboxylic acid (C75) and 9-xanthenylacetic acid (XAA) inhibited the proliferation of HCC in cell culture and in vivo at therapeutic doses [128]. The LPA-induced epithelial–mesenchymal transition (EMT) phenotype in hepatocellular carcinoma is maintained through PI3K/mTOR signaling [125]. Crosstalk between EGFR and LPARs in liver cancer has been discussed in a previous review [129].

### 4.8. Lung Cancer

LPA receptors have been reported to play a role in chemoresistance in lung cancer. LPAR1 stimulates cell growth in an ADAM12-mediated manner in A549 lung cancer cells [130]. These cells also express LPAR2 and LPAR3 receptors. LPAR1 and LPAR2 receptors are responsible for increased motility in A549-R10 lung cancer cells [131]. The authors demonstrated that in long-term cisplatin- and 5-fluorouracil- treated cells (A549-CDDP and A549-5FU), increased motility was reduced upon treatment with LPAR1/LPAR3 antagonists. LPAR3 knockdown elevated A549 survival rates in response to CDDP [131]. Aberrant LPAR5 mRNA expression due to DNA methylation in RLCNR and RH777 lung cancer cells was seen to contribute toward enhanced proliferation [132]. This group also reported that overexpression of LPAR3 attenuated migration in the same cells [133].

### 4.9. Colon Cancer

The roles of LPA and LPARs 1–6 in colorectal cancer and in the GI tract were recently reviewed [134]. Interestingly, the author points out that LPA (which is present in some foods and is generated during mastication) plays an important role in wound healing in the GI tract but can also promote tumor cell proliferation in the same tissues. A “balance” in activities is clearly important, which is logical, considering that the intestinal epithelium is continually proliferating and differentiating. This point is further emphasized in a study addressing the effects of LPA on the proliferation and differentiation of intestinal epithelial cells in organoid culture, as mediated by LPAR1 via the ERK pathway [135].

Beck and colleagues [136] reported that LPAR2 knockdown can reduce basal cell invasion in HCT116 and that an LPAR2 antagonist had similar effects. In a study using three colon cancer cell lines (Caco-2, NT-29, and HCT-118), Leve and co-workers [137] showed that LPAR1, LPAR2, and LPAR3 were expressed to varying degrees in each cell line, but that only HCT-118 cells responded to LPA with proliferation. LPA activated Rho in HCT-118, and a ROCK inhibitor blocked the proliferative response. LPA also stimulated tyrosine phosphorylation of STAT-3, and a STAT inhibitor decreased LPA-induced proliferation. Results of a transcriptome analysis indicated that LPA regulates cyclin expression through the Rho-ROCK and STAT3 pathways. 

One group performed a thorough analysis of the role of EGFR transactivation in LPA-induced proliferation and migration using a panel of six pancreatic and colorectal cancer cells [138]. In some of the cell lines, a pharmacological inhibitor of EGFR activity inhibited LPA-induced phosphorylation of EGFR, Akt, and ERK as well as DNA synthesis and migration. There was no effect of the EGFR inhibitor on DNA synthesis and migration in other cell lines, despite evidence of EGFR transactivation. The authors concluded that although LPA does transactivate EGFR in colon cancer cells, EGFR-independent pathways also contribute to LPA-induced proliferation and migration. The same investigative team came to a similar conclusion in a study of oral squamous carcinoma cells [139]. 

A recent report revealed that Agpat4, an acyltransferase that converts LPA into phosphatidic acid, is upregulated in colorectal carcinoma and is predictive of poor survival [140]. This seems contrary to the mitogenic role for LPA in colon cancer cells, but the investigators showed that LPA also promotes macrophage activity in the tumor environment to suppress tumor growth. This finding again emphasizes the spatial complexity of LPA signaling in cancer and the need for an appropriate “LPA balance.”

### 4.10. Glioblastoma

A recent study highlighted an interplay between glioblastoma cells and microglia, macrophages residing in the central nervous system [141]. The authors showed that LPAR1 antagonists inhibit microglial-induced glioblastoma cell proliferation and migration. LPA-induced G_i_ signaling has also been observed to stimulate the Rho/ROCK cascade, which plays an important role in cell motility [142]. LPA stimulates PKCα–progesterone receptor (PR) interaction inducing receptor phosphorylation, and LPAR1-mediated upregulation of VEGF expression contributing toward glioblastoma progression [143]. CD133+ U87 cells have shown enhanced responsiveness to LPA and S1P and increased ERK activation when treated with LPA. The role of LPA signaling as a therapeutic target in glioblastoma has been reviewed [144].

### 4.11. Other Cancers

The discussion above has focused on selected cancers, but these are not the only types of tumors in which LPARs are important. As recent examples, LPAR2 mediates cell survival in fibrosarcoma cells via G_12/13_ and G_i_ [145]. In bladder cancer cells, LPA promotes invasion and induces Recepteur d’origine Nantais (RON) expression [146]. LPA has been implicated in chemotherapy resistance in clear cell renal carcinoma [147]. In a gastric cancer cell line, LPA stimulates migration, invasion, and EMT via LPAR2 [148]; the investigative team also demonstrated physical interaction between LPAR2 and Notch1.

## 5. Roles of Arrestins in LPA Signaling

Beta-arrestins attenuate GPCR signaling, direct the receptors toward clathrin-dependent endocytosis, and serve as signaling scaffolds [149]. β-Arrestins also serve as scaffolding proteins that associate with signaling complexes (e.g., MAP kinases; Src). It has been clear for some time that β-arrestins regulate many classes of cell surface receptors, in addition to GPCRs [150]. These discoveries have supported the exploration of biased GPCR ligands that preferentially activate arrestin-mediated pathways [151,152]. In addition, β-arrestins have themselves been proposed as potential therapeutic targets [153].

Agonist stimulation of GPCRs leads to their internalization and results in either receptor downregulation or desensitization [154]. GRKs phosphorylate agonist-occupied GPCRs on serine/threonine residues that are exposed to the cytoplasm [155,156]. When this response was investigated in HeLa cells stably transfected with LPAR1, the results indicated that LPAR1 receptors are internalized in a clathrin- and β-arrestin-dependent manner [157]. 

The following examples support roles for β-arrestins in LPAR-mediated responses. Many groups have reported that expression levels of LPA receptors (LPAR1, LPAR2, and LPAR3) and/or β-arrestins are elevated in advanced stages of breast cancers [158]. Knockdown of β-arrestin-2 inhibited the ability of LPA to enhance migration and invasion in MCF-10A cells overexpressing LPAR1. LPAR1 enhances invasion of MDA-MB-231 cells via β-arrestin/Ral in both two-dimensional and three-dimensional assays [158]. β-Arrestins 1 and 2 can bind to ΙκΒα, the inhibitor of NF-kB [159]. LPA-induced activation of NF-kB is inhibited in β-arrestin 2-deficient cells [160]. The role of LPA in regulating the tumor microenvironment has been reviewed [161]. One group showed that β-arrestin 1 regulates the spatial distribution of actin and actin regulators, leading to RhoC-mediated invadopodia formation in various cancers [162,163] β-Arrestins have been emerging as critical regulators in cancers, making them potential targets [163,164]. β-Arrestin 2 recruits the E ubiquitin-protein ligase Mdm2, which leads to further ubiquitylation of the LPAR [165]. 

Our group examined the role of β-arrestin 2/arrestin 3 in LPA signaling in PC-3 and Du145 human prostate cancer cells [49]. In these cells, LPA-induced proliferation is mediated by LPAR1 as shown by knockdown experiments. Knockdown of β-arrestin 2 blocked the ability of LPA to induce proliferation and migration in both cell lines, indicating that these responses to LPA are arrestin-mediated. β-Arrestin 2 knockdown also blocked responses to EGF; the implications for mutually positive crosstalk between LPAR1 and EGFR are discussed in the published manuscript. This appears to be one of the few reports directly addressing the role of β-arrestins in LPA signaling in cancer cells.

## 6. Molecular Modeling of LPA Receptors

Soond and Zamyatnin [166] have emphasized the importance of using structural information to identify new targets in GPCR-mediated signaling in cancer. The Parill group was the first to publish a molecular model of LPAR1 [167]. These investigators were interested in amino acids conferring agonist specificity between LPA and the related S1P receptors. In further work, Valentine, and colleagues [168] performed the modeling of LPARs 1–3 and then did mutagenesis studies to identify the residues involved in agonist responses to both LPA and S1P.

The crystal structure of LPAR1, bound to the antagonist ligand ONO-9780307, was reported by Chrencik and co-workers [169]. They compared the model of LPAR1 to that of the sphingosine 1-phosphate receptor S1P1, since both the ligands and the receptors for LPA and S1P have structural similarities. Their results suggested that LPA (an amphiphilic molecule) accesses LPAR1 from the extracellular space, while S1P is more likely to gain access via the plasma membrane. Autotaxin has been implicated in delivering extracellular LPA to its receptors, as reviewed by Blaho and Chun [170]. These authors, reviewing subsequent work, also point out that the situation is different for LPAR6 where LPA enters the receptor binding site from the lipid bilayer. Since many lipophilic GPCR ligands access their receptors via the membrane [171], further analysis of the mode by which LPA accesses its LPAR binding site is essential for considering the actions of LPA produced extracellularly vs. within the plasma membrane. 

Chrencik and colleagues [169] also used molecular modeling to explore the ability of cannabinoid receptor ligands to interact with both LPAR1 and S1PR1, since cannabinoid receptors are structurally related to LPA and S1P receptors. The possibility that phosphorylated cannabinoid receptor ligands can bind to LPARs was discussed in this paper.

A report published in 2015 identified Lys39 in LPAR1 as critical for agonist binding; mutagenesis studies were included in this analysis [172]. In the same year, another group performed homology modeling of LPARs 1–3 with agonist and antagonist docking [173]. 

Taniguchi and colleagues [174] published a structural model of LPA6, one of the “non-EDG” members of the LPAR family. Molecular modeling of LPARs has provided considerable insight into ligand binding sites; this information is critical for drug development [175]. However, there has been little attention to date on other portions of the receptor that might be involved in protein–protein interactions, or to sites that are potentially susceptible to positive or negative allosteric interventions. The need for selective and/or biased LPAR ligands has been highlighted by Archbold and co-workers [176].

## 7. Interactions between LPARs and Other Receptors

The preceding discussion has brought forward examples of other membrane receptors (EGFR; CD97) that partner with LPARs to enhance LPA action. Spiegel’s group showed that LPAR1 and EGFR act in concert to mediate the upregulation of sphingosine kinase 1 (SphK1), which promotes invasion and motility in gastric carcinoma [177]. The same was true of oral squamous carcinoma cells where stimulation of LPAR3 led to activation of PKC and transactivation of EGFR, increasing migration of E10 cells. However, in another report [139] transactivation of EGFR was not essential for LPAR3-mediated responses in SCC-9 cells. These results suggest that further studies should be performed to clarify the crosstalk between LPARs and EGFR in cancer cells. 

Our group identified a negative interaction between an LPAR and another GPCR. We observed that the activation of free fatty acid receptor 4 (FFAR4) by omega-3 fatty acids or pharmacological agonists resulted in loss of mitogenic responses to LPA and EGF in prostate, breast, and ovarian cancer cells [59,74,178]. In prostate cancer cells, antagonist and knockdown experiments demonstrated that LPAR1 was the primary LPAR involved. Subsequent investigations showed that LPA antagonists inhibited EGF response, and that knockdown of β-arrestin 2 blocked responses to both LPA and EGF [49]. Taken together, these studies suggested that complex positive and negative interactions exist between LPARs, other GPCRs, and EGFR that cannot be easily explained by activation of downstream signaling pathways. Meizoso and colleagues [179] pursued this idea, showing that FFAR4 functionally desensitizes LPAR1. Importantly, they provided evidence that FFAR4 agonists induce a physical interaction between FFAR4 and LPAR2.

## 8. Future Directions and Remaining Questions

In the last few decades, LPA and its receptors have been studied in many different cancers. The plenitude of ways in which GPCRs interact with other proteins, including scaffolding and signaling complexes, are an increasing focus not only in cancers but in other physiological and pathological conditions. β-Arrestins have emerged as playing a vital role in the signaling mechanisms of GPCRs in cancer. The relatively few studies examining the role of β-arrestins in LPA signaling have unveiled the potential importance of arrestins. Recent crystallization studies revealed that phosphorylated serine or threonine residues in GPCRs serve as a “bar code” for β-arrestins that are required for biased agonism. LPAR1 and S1PR1 have the required barcodes, while LPAR6 contains no full bar codes which may result in the differential affinity and recruitment of β-arrestins, leading to biased signaling [170]. The development of pharmacological agents targeting LPARs has been ongoing for more than 20 years [78,175,180,181,182,183,184,185,186,187,188,189,190,191]. However, there has been relatively little exploration of the possibility of developing biased ligands for LPARs. Such ligands can potentially fine-tune LPAR response in a therapeutic direction, particularly in cancer signaling.

**Figure 2:**
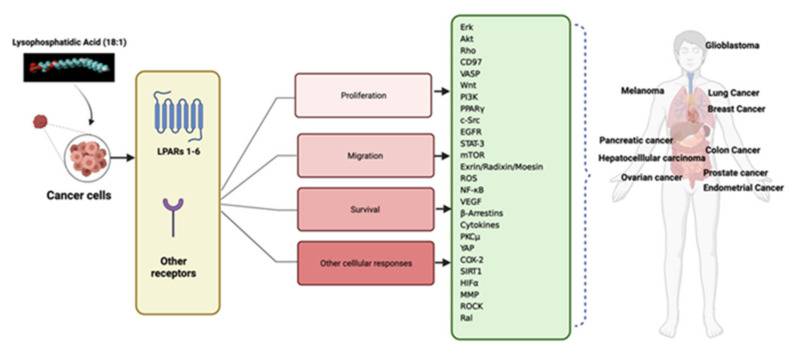
Lysophosphatidic acid responses in human cancers. LPA binds to LPARs 1–6 as well as to other non-GPCR receptors. These receptors can mediate the proliferation, migration, and survival of cancer cells as well as other tumor phenomena such as angiogenesis and progression. The green box lists signal transduction events, discussed in this review, that occur downstream of LPAR activation. These signals contribute to the cancers that are shown in the human figure and are specifically discussed in this review. This figure was made using www.biorender.com; Accessed on 4 August 2021.

The analysis presented herein suggests several answers to the question of what makes LPA unique as a GPCR agonist in cancer cells. First, the multiplicity of LPA receptors is unusual, even for GPCRs. It is clear from the literature that most cells express more than one LPA receptor and that pro-tumorigenic activities have been attributed to most or all these receptors when they are expressed in cancer cells. The different LPARs are coupled to different G proteins, and when activated, they initiate distinct but sometimes overlapping cellular responses. The profile of LPAR expression varies between types of tumors, and between cell lines derived from these tumors. Knockdown of a single LPAR may not eliminate a given response, because there can be redundancy of receptors initiating that response. In addition, the non-GPCR receptors for LPA (PPARγ, etc.) add to the plethora of potential responses. There is no single outstanding feature of LPAR-mediated response pathways. However, the sheer complexity of LPA response, even within a single cell, is likely part of what makes LPA special. Figure 2 summarizes the signaling elements discussed in this review.

Second, LPARs engage in crosstalk with other receptors, including EGFR. This further expands the range of downstream signal transduction and provides opportunities for negative modulation as well (e.g., with FFARs). These crosstalk events are not exclusive to LPARs but appear to be particularly prominent aspects of LPA response. In a general sense, it seems likely that receptor-specific aspects of GPCR signaling involve protein–protein interactions, since such interactions are part of the “signature” of a given receptor. In other words, there are more possibilities for “uniqueness” inherent in the tertiary structure of the GPCR than in any combination of canonical G protein and arrestin-mediated signaling responses. 

Third, it is important to keep in mind that LPA refers to a family of lipid mediators with different substituents and linkages; the physiological milieu contains many LPA species. Studies conducted in cell culture typically use 18:1-LPA. The activity and binding profiles of other forms of LPA have been examined over the years [19,46,184], but a more comprehensive understanding of LPA species (e.g., additional studies of ligand selectivity of different LPARs, potential signaling bias of different LPAs, computational modeling of LPA–LPAR interactions, and LPA species profiles in different tissue microenvironments) would be informative.

There are several gaps in knowledge in this research area that deserve further attention. First, the elegant, published work in molecular modeling of LPARs has not yet addressed regions of the proteins that may interact with other signaling partners (e.g., EGFR, CD97, FFARs). These regions represent sites for potential modulation by ligands acting allosterically. The extent to which LPARs dimerize with other LPARs, or with other GPCRs, is currently not clear. Second, the contribution of EGFR transactivation to overall LPA response has been investigated in only a few model systems. It is important to know whether this is the major pathway for LPA-induced proliferation in cancer cells. The molecular basis for the transactivation is not yet completely clear. While the proteolysis-mediated release of EGFR ligands is an appealing paradigm with much experimental support, the potential for direct interaction between LPARs and EGFRs remains relatively underexplored [102]. Finally, the roles of arrestins in attenuating and/or propagating LPA signaling in cancer are surprisingly understudied, even though this topic is being addressed in other disease states [192]. Since β-arrestins appear to mediate many of the prolonged actions of GPCR agonists, including cell proliferation, their role in LPAR response needs to be considered. This is especially the case since arrestins can act as scaffolds for protein–protein interactions, including those with other receptors including EGFR. 

In conclusion, LPARs present a fascinating range of signaling complexity that will be unraveled over time. In the words of a sage physician-scientist, “In the last five years more interest has been shown … and if this continues for another ten years, we may have an understanding of the subject” [193]. The topic of LPA in cancer has been well studied in many respects, but much remains to be investigated. In many ways, LPARs serve as examples for other GPCRs that can stimulate cell proliferation. GPCRs hold promise as signal transduction targets in cancer therapy, with the hope of modulating tumor cell growth without cytotoxicity to normal cells. It is an ever-evolving subject when investigating molecular mechanisms in cancer.

## Data Availability

Not applicable.
